# Non-streptomycete actinomycetes nourish the current microbial antibiotic drug discovery

**DOI:** 10.3389/fmicb.2013.00240

**Published:** 2013-08-20

**Authors:** Polpass Arul Jose, Solomon Robinson David Jebakumar

**Affiliations:** Department of Molecular Microbiology, School of Biotechnology, Madurai Kamaraj UniversityMadurai, India

**Keywords:** actinomycetes, rare actinomycetes, *Streptomyces*, non-streptomycetes, antibiotics

Antibiotic drug discovery is an indispensable process to combat aggressive ability of pathogenic microorganisms and emerging infectious diseases against health and well-being of people throughout the world. According to the updated and expanded data on natural products presented by Newman and Cragg (2012), the utility of natural products as sources of novel structures is still alive and the anti-infective area depends on natural products and their structures. The natural products with pharmaceutical importance are largely produced from primary and secondary metabolism of plants and microorganisms. Microbial natural products have made an incredible contribution to the antibiotic drug discovery and development process over last seven decades (Demain and Sanchez, 2009). Alexander Fleming's discovery of penicillin from a mold in 1928 and its subsequent progress into a medicine in the 1940s laid the foundation for development of microbial natural products as drugs (Fleming, 1929; Chain et al., 1940). Later, Waksman's report of actinomycin in 1940 and subsequent reports of streptothricin in 1942 and streptomycin in 1943 introduced the actinomycets as source of antibiotics (Waksman and Woodruff, 1940; Waksman, 1943; Comroe, 1978). In the end of 20th century, several actinobacterial natural products had found extensive applications in clinical field as antibacterial, antifungal, antiparasitic, and anticancer agents (Butler, 2004). Of all the reported bioactive compounds of microbial origin, 45% are produced by actinomycetes (Berdy, 2005). Moreover, 90% of practically used antibiotics are originated from the actinomycetes (Hamaki et al., 2005) and they are still being the chief natural antibiotic producers.

Since the report of streptomycin from the strains of *Streptomyces griseus* there has been a rapid escalation in antibiotic discovery from the genus *Streptomyces* (largest genus of Actinobacteria). Subsequently, members of this genus have become renowned as a prime source of natural antibiotics (Mohamed and Galal, 2005; Jose et al., 2011). It has been evidenced by continuing production of string of commercially important antibiotics like daptomycin, erythromycin, fosfomycin, lincomycin, neomycin, streptomycin, and tetracycline from industrially-important members of the genus *Streptomyces* (Mahajan and Balachandran, 2012). However, the likelihood of finding novel antimicrobial leads from these distinctive actinomycetes has recently dwindled upon the rediscovery of known compounds (Koehn and Carter, 2005) and the focus of current microbial drug discovery programs being reoriented toward other promising microbial resources. Other side of the coin, emergence of new infectious diseases and antibiotic resistance provoked in earlier infectious diseases has raised the need for novel antibiotics.

Against this backdrop, rare actinomycetes (non-streptomycetes) are currently isolated from diverse environments and intensively studied for potential leads for antibiotic discovery programs. The rare actinomycetes are usually regarded as strains of actinomycetes whose isolation frequency by conventional methods is much lesser than that of streptomycete strains (Seong et al., 2001). According to a recent review by Tiwari and Gupta (2012), this non-streptomycete actinomycetes group comprising diverse bioactive secondary metabolite producing members under following genera: *Actinomadura, Actinoplanes, Amycolatopsis, Dactylosporangium, Kibdelosporangium, Kitasatospora, Microbiospora, Planomonospora, Planobispora, Salinispora, Streptosporangium*, and *Verrucosispora*. The list has further been extended by recent reports of bioactive compounds from members of other rare genera, *Nonomuraea* (Beltrametti et al., 2003; Jose and Jebakumar, 2012; Flatt et al., 2013), *Actinoalloteichus* (Fu et al., 2011; Wang et al., 2013), *Pseudonocardia* (Oh et al., 2009; Barke et al., 2010; Carr et al., 2012), *Saccharothrix* (Murakami et al., 2009; Aouiche et al., 2012; Nakae et al., 2013), and *Actinosynnema* (Wei et al., 2010; Siyu-Mao et al., 2012). Moreover, basic knowledge of the habitats, physiology, and secondary metabolite diversity of the rare actinomycetes gradually increased (Tiwari and Gupta, 2012). Out of more than eight thousand antimicrobial products described in the ABL database, 16% produced by strains belong to rare genera of actinomycetes(Lazzarini et al., 2001).

Distribution of the rare actinomycetes has been found to be wide in terrestrial and aquatic environments (Tiwari and Gupta, 2012). Fair isolation methods have become indispensable as these rare actinomycetes exert slow growth while fast growing streptomycetes and fungal strains dominate in normal actinomycetes isolation methods. Different and isolation methods have to be employed for acquiring diverse actinomycetes from different sources. These methods employ variety of pretreatment techniques and enrichment techniques along with specific isolation medium amended with specific antimicrobial agents (Hayakawa, 2008). Tiwari and Gupta (2013) have reviewed almost all the selective isolation methods that have been developed to date for isolation of rare actinomycetes. In fact, all the isolation methods are designed to favor the growth of rare actinomycetes while suppressing the growth of undesired microorganisms.

In recent times, much attention has been given to isolate rare actinomycetes from diverse previously unexplored common as well as uncommon extreme environments. Numerous rare actinomycetes have been isolated from variety of soil samples (Seong et al., 2001; Gu et al., 2007; Li et al., 2007, 2010; Ara et al., 2013), plant materials (Qin et al., 2009; Janso and Carter, 2010; Zhao et al., 2011), marine sources (Zhang et al., 2006; Sun et al., 2010; Radhakrishnan et al., 2011; Goodfellow et al., 2012), extreme saline zones (Jose and Jebakumar, 2013), Volcanic zones (Lee and Lee, 2011) hyper arids (Okoro et al., 2009), glaciers (Reddy et al., 2010; Zhang et al., 2012), and much more. As a result, an array of rare actinomycetes available for current antimicrobial drug screening programs has increased. A comprehensive recent review by Tiwari and Gupta (2013) summarizes the reports of isolation rare actinomycetes from different natural habitats throughout the world. It is disclosed that, an unexpected variety of rare actinomycetes populates in diverse, previously unexplored natural habitates.

In conclusion, rare actinomycetes are being unveiled as highly prospective sources of bioactive compounds. Paths have already been sketched to reach these rare microorganisms but strides should be taken with specific isolation strategies. The progressing discoveries of novel bioactive compounds from these rare actinomycetes (non-streptomycetes) apparently opine that these organisms nourish the current antibiotic drug discovery programs.
